# Scientific Mapping of Gamification in Web of Science

**DOI:** 10.3390/ejihpe10030060

**Published:** 2020-08-20

**Authors:** Jesús López-Belmonte, Mª Elena Parra-González, Adrián Segura-Robles, Santiago Pozo-Sánchez

**Affiliations:** 1 Department of Didactics and School Organization, University of Granada, Ceuta 51001, Spain; jesuslopez@ugr.es (J.L.-B.); santiagopozo@correo.ugr.es (S.P.-S.); 2 Department of Research Methods and Diagnosis in Education, University of Granada, Ceuta 51001, Spain; adrianseg@ugr.es

**Keywords:** gamification, active methodology, educational innovation, bibliometric analysis, scientific mapping

## Abstract

Education is a constantly changing field. The new teaching processes are developed today and all teachers should be prepared. Gamification is one of the methodologies with the greatest impact on the learning process. The objective of the study is to analyze the relevance and progression that the concepts “gamification” and “learning” have acquired in the scientific literature of Web of Science. This research has been based on a bibliometric methodology. A classic bibliometric and thematic analyses have been carried out. A 1230 document matrix analysis has been selected. For descriptive bibliometrics, the Bibliometrix library is used, while Scimat is used for the thematic analysis. Both tools are widely used in this type of study. Results indicate that growth on the study of these topics is booming. There are also authors who accumulate most of the documents, such as Martí Parreño. English is positioned as the predominant language. The topics studied have evolved from how classic games affect learning to the study of video games and their impact on performance. It is concluded that these types of studies are still relevant and with a great future prospect. Furthermore, research focuses especially on video games, on the effects of technology on learning and on the study of flipped learning experiences.

## 1. Introduction

The inclusion of information and communication technologies (ICT) in the educational field has changed the way of conceiving the teaching and learning process [1]. In this way, technological devices are part of the daily life of students within the educational context [2] and the daily life of society in general [3]. This socio-educational panorama has brought about important changes in the configuration of the teaching and learning processes of the students [4]. Educational centers have incorporated technological resources into pedagogical methodologies [5] to streamline educational contexts [6] and promote student motivation through the work of digital competence [7].

Therefore, the use of ICT in student learning is essential in today’s education [8,9] so that pedagogical processes adapted to the digital age can be carried out [10]. Current education should aim to implement methodologies that boost the instructional process [11] and generate new learning experiences [12] based on the potential of virtual spaces and technological devices [13].

Along these lines, new pedagogical approaches, methodologies and learning techniques based on achieving these objectives have proliferated in recent years. Among the multitude of pedagogical possibilities that currently exist, gamification stands out, a learning technique focused on applying the dynamics, structures and rules of the game in formal learning contexts [14]. This learning technique is born outside the academic environment, since its origin is in the business context as a dynamic to achieve customer loyalty [15]. Under the premise of considering play as a fundamental element in the optimal development of children, this technique was transferred to the educational field [16].

In this way, a learning technique based on transferring the dynamics of the game to the pedagogical field was created [14,17] in order to improve the results of the students, promote the acquisition of knowledge dynamically, improve their skills or reinforce actions specific that promote the competence development of the student [18]. The implementation of gamification in the classroom fosters important values for the development of students [19] by carrying out a restructuring of the usual grading system in traditional education and complementing it by reward systems [20]. In this way, the student takes the leading role in their own training in a playful environment in which learning appears competitively and with high levels of motivation [21].

Many authors have found that the inclusion of gamification in the pedagogical field causes many benefits [22]. The main benefit is related to learning motivation, since gamification presents tasks and activities in an attractive way for students [23,24]. Likewise, it also encourages students to improve their ability to solve problems [25], their commitment [26,27] and their participation [28,29]. The interest of the students increases exponentially during the implementation of the gamification, in such a way that the achievement of fundamental competences for their development is fostered [30], in an environment that encourages the correct behavior of the students for compliance with the rules [31,32] as well as an important development of social skills [33]. Likewise, gamification has the advantage of being used as an imbricate pedagogical strategy in a multitude of methodologies, especially those that promote the active role of students and that give ICT a leading role in the teaching and learning process [34,35].

This study will expand the available bibliometric information on gamification in educational contexts, a field of research that has few reported studies [36,37,38,39]. For all the above, it is necessary to analyze the existing scientific production on a field of research with high production rates in recent years, especially due to the emergence of gamification as a learning technique increasingly used among the teaching community. The results achieved here will serve as a starting point for future work and to offer the scientific and teaching community the state of the art on gamification in learning environments.

In this paper, the concepts of "gamification" and "learning" are analyzed from a bibliometric perspective of the existing literature on these terms. In this case, the aim was to analyze the relevance and progression that the concepts “gamification” and “learning” have acquired in the scientific literature of Web of Science (WoS) database. This database has been chosen because it is one of the main repositories of scientific production worldwide. Furthermore, experts in this type of analysis choose to analyze this database for presenting a greater literary volume compared to other databases [40,41,42]. In addition, this database contains a large volume of studies related to the social sciences, the field of knowledge related to gamification and learning [43].

The originality of this study focuses on revealing to researchers the new findings of the analyzed constructs by means of a novel document analysis technique. The work presented is distinguished from other studies by the type of research design carried out. Previous studies [36,37,38,39] have focused on analyzing the state of affairs using classical production-centered bibliometric methods. Specifically, in this research a scientific mapping has been used that has allowed the dynamic and structural evolution of the defined terms to be carried out. This technique has not been previously used to analyze the selected constructs, so an exploratory and novel study in the impact literature is presented. This work has started from the analytical premises and procedures of previous impact studies that have followed this innovative documentary analysis technique [44,45].

The purpose of this study is to analyze the relevance and progression that the concepts of "gamification" and "learning" have acquired in the WoS scientific literature. To the best of our knowledge, no study of this nature has been reported. For this reason, this work is carried out with an exploratory component. In addition, this research will contribute to reducing the gap found in the literature on these terms and increasing the body of knowledge on the subject. This topic is highly projected in the literature but has not been sufficiently addressed from a bibliometric perspective such as the one carried out in this study. All this will serve as a starting point for other investigations by the research community. Therefore, this study pursues the following objectives:

- To know the performance of scientific production indexed in WoS on “gamification” and “learning”.
- To concrete the scientific evolution in WoS on “gamification" and “learning”.
- To discover the most important issues in WoS about “gamification" and “learning”.
- To determine the most incidental authors in WoS about “gamification" and “learning”.
- To analyze collaboration networks.

In addition to the specific objectives, the following research question is formulated to specify the path towards the achievement of the previous statements:

- What is the path that gamification has had in the field of learning?
- What are the gamification trends in learning environments?

## 2. Materials and Methods

### 2.1. Research Design

Bibliometry has been used as the research methodology. Among its potential are the actions of search, registration, analysis and prediction of the scientific literature [46]. Therefore, to achieve the objectives formulated and to effectively carry out this study, the precepts of experts in this field of science have been followed [47].

In turn, this research has focused on an analysis of co-words [48] and of various indices of a scientific nature such as the h, g, hg and q2 index. The use of these bibliometric indicators is based on various impact studies reported from prestigious and scientifically relevant databases [49,50,51].

The research design carried out has derived in the preparation of maps with nodes to delimit the performance and location of the conceptual subdomains related to gamification and learning [52]. Similarly, these analytical actions have led to the presentation of the thematic development of the terms mentioned in the WoS publications [53].

### 2.2. Procedure and Data Analysis

In order to carry out an accurate investigation and minimizing the possibility of the appearance of biases, the study was carried out using the following procedure:

1. Choose the database for documentary analysis (WoS).
2. Select the keywords (“gamification” and “learning”).
3. Construct the search equation (“gamification” AND “learning” [TITTLE] in the categories of “Education Educational Research”, “Education Scientific Disciplines” and “Psychology Educational”).
4. Perform the search action in the title, abstract and keywords metadata of the documents registered in WoS.

These deployed actions produced a total of 1220 publications. To refine the document search and reporting process, different criteria have been established to include and exclude publications. The following have been defined as exclusion criteria: documents published in the year 2020 (for not having finished the year); repeated or poorly indexed documents in WoS.

**Figure 1:**
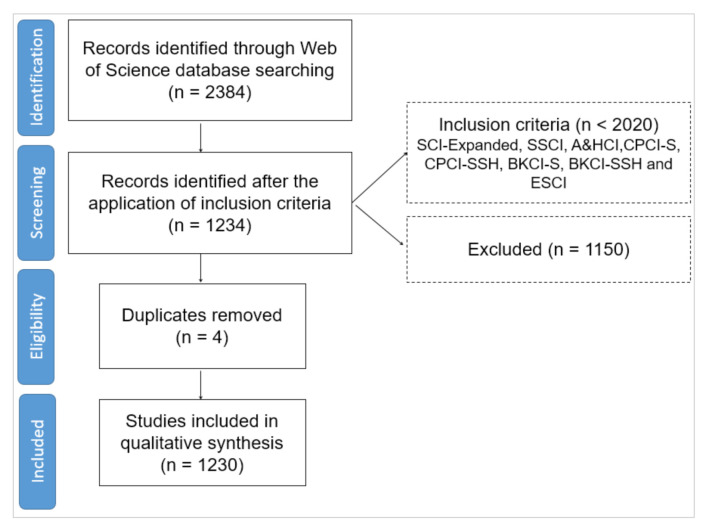
Flow diagram based on the protocols of the PRISMA-P matrix. Databases used: Science Citation Index Expanded (SCI-Expanded), Social Sciences Citation Index (SSCI), Arts and Humanities Citation Index (A&HCI), Conference Proceedings Citation Index—Science (CPCI-S), Conference Proceedings Citation Index—Social Science and Humanities (CPCI-SSH), Book Citation Index—Science (BKCI-S), Book Citation Index—Social Sciences and Humanities (BKCI-SSH) and Emerging Sources Citation Index (ESCI).

All this procedure has been reflected in the following flow diagram based on the Preferred Reporting Items for Systematic review and Meta-Analysis Protocols (PRISMA-P) matrix (Figure 1). This protocol is one of the most widely used within the Bibliometrics scientific community. It consists of a set of guidelines that make up a seventeen-element checklist that contributes to setting up an effective protocol for systematic review.

The documentary analysis has been carried out using three software applications [54]. Specifically, Analyze Results, Creation Citation Report (tools integrated into the WoS platform) and Bibliometrix have been used to extract and analyze the data regarding the year, authorship, country, type of document, institution, language, medium and most cited documents.

**Figure 2:**
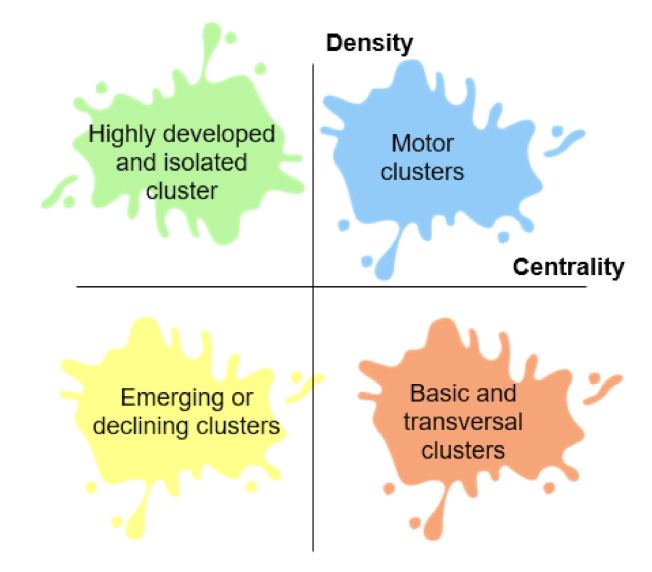
The strategic diagram interpretation.

The tools integrated into the WoS platform were used, on the one hand, for their ease of use and, on the other hand, for the veracity and relevance of the results obtained, which allows reducing the margin of error intrinsic to the use of tools external to the platform. Bibliometrix is an open source resource to carry out quantitative bibliometric research that allows obtaining bibliographic data from the main scientific databases, constructing citation matrices, co-word analysis and collaborative analysis. Furthermore, the Science Mapping Analysis Tool (SciMAT) has been used to analyze the structural and dynamic development of publications on "gamification" and "learning" from a longitudinal aspect. This tool is an open source software that allows to analyze scientific maps longitudinally and helps researchers to process and refine bibliographic data, use bibliographic measurements and configure the analysis for scientific mapping. The researchers have followed the recommendations of the experts for the proper use of the aforementioned programs [55], since its use is adjusted to the needs of the research and allow to specifically achieve the achievement of the objectives formulated for this study. Especially, with SciMAT different actions have been carried out to carry out the analysis of co-words [56]:

- Recognition: different actions took place in this process: (1) the keywords of the WoS indexed publications on the subject were analyzed (n = 3721); (2) co-occurrence node maps were designed; (3) a standardized network of co-words was created and the most significant keywords (n = 3524) were delimited; and (4) the most influential topics and terms were established with a clustering algorithm.
- Reproduction: in this process, different thematic networks and strategic diagrams were generated (respecting the principles of centrality and density) made up of four zones (Figure 2) (upper right = relevant and motor themes; upper left = rooted and isolated themes; lower left = disappearing themes or in projection; lower right = issues of low development and cross-sectional).

- Determination: the volume of documents reported has been classified in different time periods. This has allowed analyzing the evolution of the nodes in different time intervals. Specifically, three periods were established (P1 = 2011–2015; P2 = 2016–2016; P3 = 2018–2019). As a configuration criterion, these intervals were set up so that each one had a similar volume of documents. To analyze the authorship of the publications, only a single period has been configured that covers the entire time span studied (P = 2011–2019). The strength of association between the intervals has been determined by the number of keywords in common between the different established periods.
- Performance: to carry out this process, various production indicators and inclusion criteria were taken into account (Table 1).

## 3. Results

**Figure 3:**
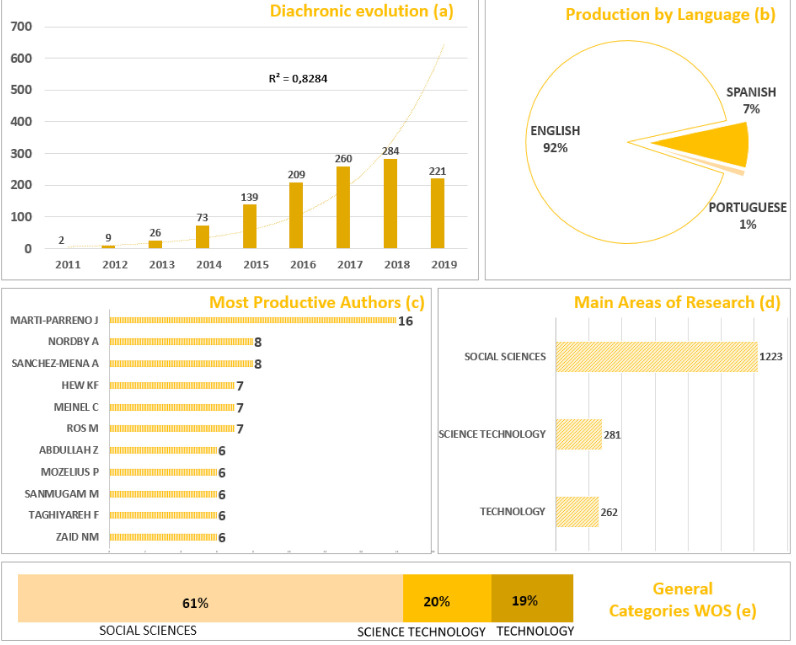

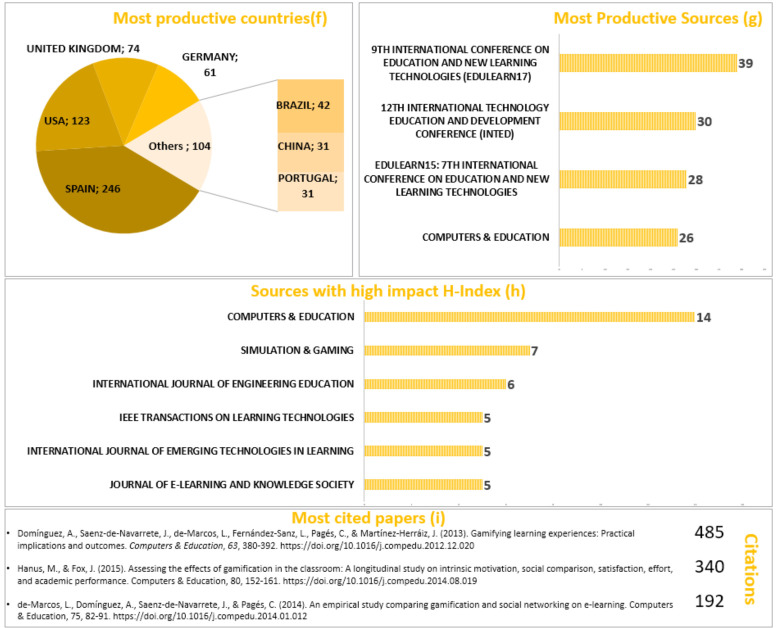
Diachronic evolution of production (**a**), the countries with the highest production (**b**), the most productive authors (**c**) and main areas of research (**d**). General categories in Web of Science (WOS) (**e**), most productive countries (**f**), most productive sources (**g**), sources with high impact based on H index (**h**) and most cited papers (**i**).

A total of 1220 publications were retrieved to final analysis. There were 1068 institutions and 3110 authors from 56 countries. Figure 3 shows the statistical results of the diachronic evolution of production (a), the countries with the highest production (b), the most productive authors (c) and main areas of research (d), general categories in WOS (e), and classical values for pre bibliometric analysis.

The growth of the literature throughout the period 2011–2019 is an exponential growth (a). The number of documents grows every year with a trend of r^2^ = 0.82. This coefficient of determination (r^2^) reflects an adequate goodness of fit with respect to the corresponding variable number of publications per year. The most important growth occurs in the period 2012–2013 tripling the number of studies. In 2019, there is a small decrease in the number of publications, normal within the classic development, produced in part by the delay of WOS in the indexing of documents. Regarding the main language of publication (b) prominently in English with 92% of the documents followed by Spanish with 7%. MARTI PARRENO is the most productive author with 16 documents (c). Finally, most documents are directly related to the social sciences both in the main areas of research (d) and in the general categories of WOS (e).

From the perspective of the countries, (f) Spain stands out with 246, followed by the USA with 123, the United Kingdom with 74 and Germany with 61. Most publications are made at conferences (g), being "9TH INTERNATIONAL CONFERENCE ON EDUCATION AND NEW LEARNING TECHNOLOGIES "which accumulates 39 publications. However, the impact of these conferences at the scientific level (h) is less than that of the journal Computers and Education, with an average H index of 14 of their publications, much higher than the rest. Finally, the most cited documents in the entire period analyzed (i) belong to the same journal, accumulating more than 800 citations in total.

### 3.1. Collaboration Networks

**Figure 4:**
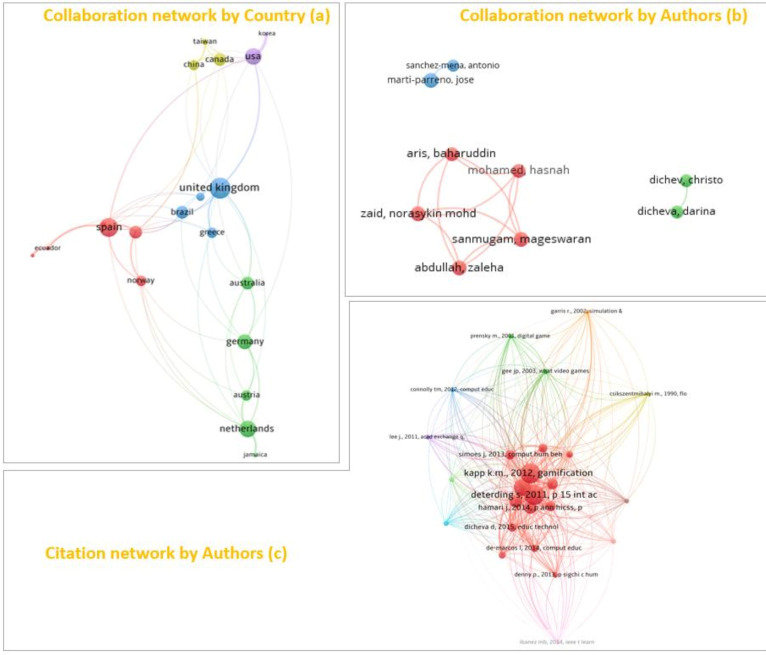
Collaboration network by country (**a**), collaboration network by Authors (**b**), citation network by Authors (**c**).

Similarly, the collaboration and citation networks of the documents that make up the work matrix have been analyzed (Figure 4). Depending on the country (Figure 4a), the countries that collaborate the most are Spain and the United Kingdom. The first of these, in red, collaborates with many Spanish-speaking countries in addition to Norway. On the other hand, the United Kingdom does it with Russia, Greece and Brazil.

Regarding collaboration between authors, it has been only found three well-differentiated groups (Figure 4b), which are shown around the red, green and blue clusters, the first being the most numerous collaboration network. Finally, the citations network is analyzed, where you can see the references most used by all the authors. This type of analysis allows us to know which are the main authors (basic bibliography) on a subject analyzed. Kapp and Detering stand out as the most cited authors in all papers, followed by Hamari and Simoes (Figure 4c).

### 3.2. Development of Thematic Analysis

For the thematic analysis, the documents have been classified into three main blocks. Those documents published between 2011–2015, 2016–2017 and 2018–2019. The reason for this division is the number of documents published for each period, trying to be consistent with its growth for the total period.

**Figure 5:**
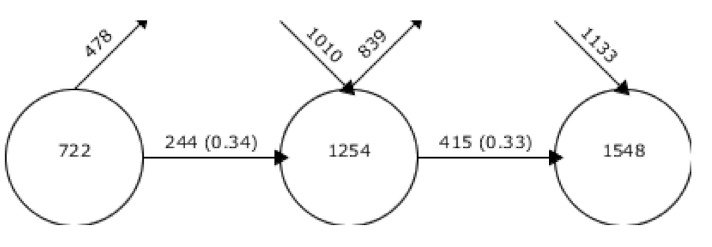
Evolution of keywords between intervals.

Figure 5 graphically shows the evolution of the keywords between the periods. The exit arrow indicates the number of keywords that are maintained in the following period while the incoming arrow indicates the new words included for the period. Between the periods, there is a consistency of keywords of 0.34 and 0.33, which indicates that it has not remained stable throughout its evolution.

Among the proposed and analyzed blocks, a series of keywords are observed for that range (Table 2). The indicators are shown based on the bibliometric indicators most used by today’s databases (h-index, g-index, q2-index) and the number of citations received. Following the H index, in the first period (P1), GAMES and LEARNING stand out, only behind the GAMIFICATION keyword. In period two (P2), PERFORMANCE stands out with a value of 16. For the third and last period (P3), INTRINSIC-MOTIVATION stands out followed by SATISFACTION, VIDEO-GAMES and IMPACT.

**Figure 6:**
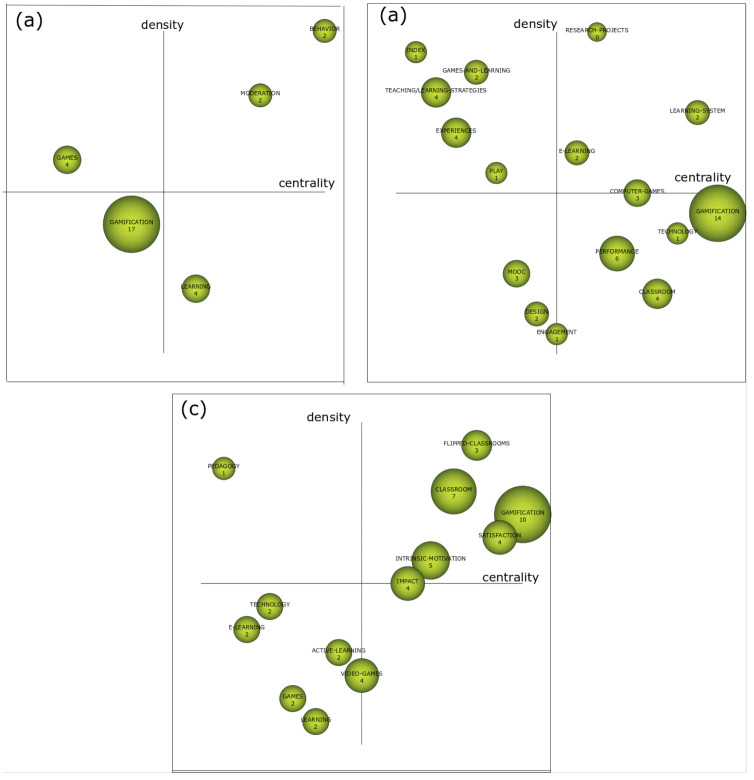
Strategic diagram of h index. (**a**) Interval 2011–2015; (**b**) interval 2016–2017; and (**c**) interval 2018–2019.

The thematic evolution networks are presented following Callon’s density and centrality measurement. This index allows us to graphically represent a co-word analysis and measure its degrees of interaction. Consequently, its use is the most appropriate for this type of study in which the graphic representation of the analysis of joint words and the interactions between them is intended using this tool, which is included in the SciMAT software. The internal strength of the network can be measured by the density defined as internal links between all the keywords that are grouped around a specific topic [57]. Figure 6 graphically shows the trends detected for each period. In the first period, the relationship between gamification and GAMES prevails as a theme, while studies mainly related to the effects on BEHAVIOR and its MODERATING effect are carried out. In the second period, there is a wide diversification of the topics analyzed. This is how TEACHING / LEARNING STRATEGIES and developed EXPERIENCES stand out as motor. The driving themes are E-LEARNING and new LEARNING SYSTEMS. For the last period, the motor themes are related to FLIPPED LEARNING, MOTIVATION and IMPACT on the learning process. For this last period, it is interesting to highlight the topics with greater prospects such as VIDEO-GAMES, ACTIVE-LEARNING and TECNHOLOGY.

**Figure 7:**
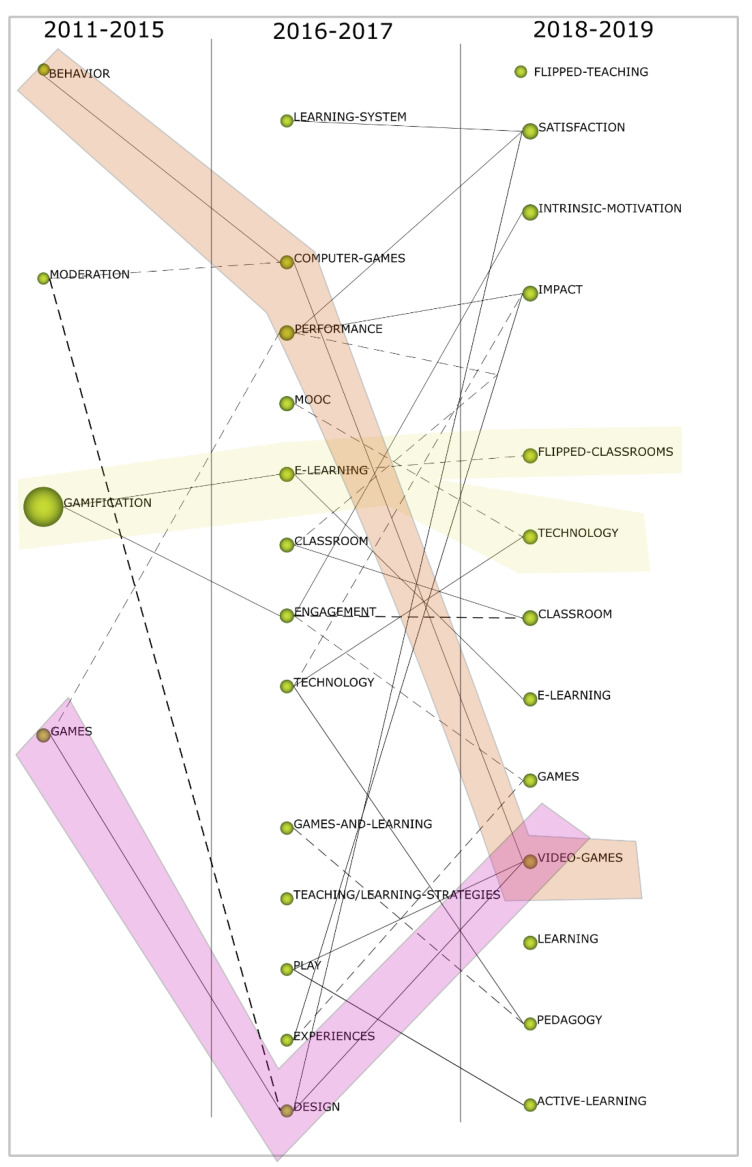
Thematic evolution of the analyzed area 2011–2019.

A thematic analysis was carried out in all the periods analyzed. The thickness of the line shows the strength of the related ones found. The size of the sphere shows the average citation counts for each of the topics analyzed. Citation count allows us to measure the importance and impact of the subject for one of several periods. Color bring indicates the labeled subjects within the same thematic area across periods. As can be seen in Figure 7, throughout the analyzed periods, the thematic variety stands out. During the three periods, three different approaches stand out. The first (orange) shows the evolution followed by studies focused on behavior towards the influence of computer games and video games on the educational process. In the second (yellow), it is observed that the studies on gamification have evolved with the rhythm of technology and have been adapting to other perspectives such as e-learning or flipped learning.

Finally, in pink it is observed how the studies have gone from studying games in general to studying their design and video games in the last period. Another interesting aspect is knowing where the topic of gamification is evolving today. The last period shows a growing trend of research related to flipped learning, e-learning and videogames. In summary, all these investigations try to study the effects of active learning as new pedagogical models and their impact on variables such as motivation or learning.

## 4. Discussion and Conclusions

This study analyzed the relevance and progression of the concepts "gamification" and "learning" within the scientific literature in WoS. So far, there were no published studies of this nature, so this research will contribute to reduce the gap within the scientific literature on these terms, thus providing knowledge on the subject. Bibliometric analysis of scientific literature is used to picture fields and areas of knowledge [58].

After having analyzed the significance and progression of the concepts “gamification” and "learning" concept in an educational context, the main results are discussed. The first document where these concepts began to be used dates from 2011.

It has been shown that more than 1000 institutions and more than 3000 authors published about these concepts along these years. From 2016 onwards, the production increased in a significant manner, being the production of more than 200 documents each year. As other studies highlighted, it has been found that English is the most publishing language, becoming this one the scientific language worldwide [51,59,60,61]. Apart from this, WOS has its own penalties when published documents are not in [14].

It should be noted that the discussion of the results obtained in this research with other publications is complicated. Although there are some papers that carry out a bibliometric analysis on gamification, these do not coincide with the periods, the databases or the research fields analyzed in this study. Despite this, it is possible to establish some similar trends regarding the bibliometric results obtained. The number of published documents on gamification in education increases exponentially, with a greater number of documents published in the last third of the century [36,37,38,39], especially between 2018 and 2019 [37,38].

A great disparity has been observed regarding the number of documents analyzed. In this study, 1220 publications were analyzed, a figure similar to that reported by a recently conducted bibliometric analysis [37], although it differs from analyzes that have analyzed different periods [36,38,39], which have obtained less documents to analyze. The justification for this aspect is related to the disparity found in the choice of exclusion criteria from the bibliometric search.

On the other hand, the scientific literature has reported that the most frequent type of document for gamification in the educational field is the article [36,37,38], despite the fact that other research highlights the importance of the conference paper as a method of publishing this thematic [37,39]. In line with the results obtained in this research, the field of research reported by other studies with greater importance within this topic is that of the social sciences [36,37,38,39], although computer sciences also stand out [36,37,39].

Likewise, this research highlights Martí-Parreño as the author with the highest production in the field of educational gamification, despite the fact that other research highlights authors such as Deterding, Dixon, Khaled and Nacke [37,39], Domínguez, Saez and Marcos [36] or Kietzmann, McCarthy, Pitt, Plangger and Robson [38], although it is necessary to consider that the periods analyzed are different.

On the other hand, the results obtained regarding the origin of the publications coincide with those obtained in this study, highlighting Anglo-Saxon countries such as the United States and England, in addition to Spain [36,37,38,39]. Despite this, the contributions made by authors from Germany are also highlighted [39].

The evolution of keywords between those years were constant at the level of production, with the percentage of keyword matching between periods, which is above 30%. This is a sign that new educational trends are being generated within this field of study.

This study has implications for both theory and practice aspects. In relation to theoretical implications, this research offers relevant information to the scientific community about gamification and learning within educative processes at several stages. First of all, this research provides the most relevant theoretical contributions from an innovative technique in the field of bibliometric researches. The documents used in the analysis are indexed in WOS, which show their relevance. Moreover, this study allows us to establish a profile of the type of documents that are usually presented about gamification and learning. From this study there is offered a clear view on the important information on the topic about the language, the publication area, the type of document, the organizations, the authors, the publication sources, the countries, and even the citations. All this information can help researchers to focus their work. In addition, this research made it possible to know the most relevant topics around the terms, and to identify the main lines of researches during last years. Finally, talking from practical implications, the results shown in this study provide relevant information for teachers and professors about gamification and learning. This is because this study can help them to take decisions in their everyday practice.

This research can serve as a starting point for future researchers and other researchers among the scientific community. The main contribution of this research is to provide to the scientific field and researchers new trends on the most relevant production and evolution of this specific topic.

Although this analysis of these periods was done in depth, there are limitations that have to be considered. First of all, WoS has its own data debugging process. Therefore, there can be small distortions which are likely to happen when working with a large number of indexed publications. Moreover, the parameters used within this study were established by the researchers, who presented the results in accordance with size and relevance and according to the established criteria. In addition, it was decided to delimit the search field with the concept of learning. The term education was not taken into account. This limited the results to that which is related to the methodology at a training level, in relation to the learning component. Finally, the implications and possible applications were based on the most important results obtained in the strongest period, which can be considered as future lines of study.

## Figures and Tables

**Table 1:** Production indicators and inclusion criteria.

Configuration	Values
Analysis unit	Keywords authors, keywords WoS
Frequency threshold	Keywords: P_1_ = (2), P_2_ = (2), P_3_ = (2)
Network type	Co-occurrence
Co-occurrence union value threshold	Keywords: P_1_ = (2), P_2_ = (2), P_3_ = (2)
Normalization measure	Equivalence index
Clustering algorithm	Maximum size: 7; Minimum size: 4
Evolutionary measure	Jaccard Index
Overlapping measure	Inclusion Rate

**Table 2:** Classic bibliometric indicators (h-index, g-index, q2-index) for analyzed periods.

**2011–2015 Period**					
	**Docs**	**h-index**	**q-index**	**q2-Index**	**Cites**
BEHAVIOR	2	2	2	14.63	89.5
MODERATION	2	2	2	14.63	89.5
GAMIFICATION	98	17	42	33.5	18.61
GAMES	9	4	6	8.94	17.56
LEARNING	5	4	4	16.97	39.6
**2016–2017 Period**					
	**Docs**	**h-index**	**q-index**	**q2-Index**	**Cites**
RESEARCH-PROJECTS	3	0	0	0	0
LEARNING-SYSTEM	5	2	3	4	3.2
GAMIFICATION	200	14	22	18.71	3.77
COMPUTER-GAMES	8	3	5	4.9	4.12
PERFORMANCE	13	6	11	10.39	11.62
MOOC	12	3	5	4.58	2.92
E-LEARNING	12	2	3	3.46	1.33
CLASSROOM	11	4	7	14.42	14.09
ENGAGEMENT	10	1	2	2.45	0.8
TECHNOLOGY	7	1	2	4.24	2.71
INDEX	2	1	2	6.24	20
GAMES-AND-LEARNING	6	2	3	4	2.17
TEACHING/LEARNING-STRATEGIES	5	4	5	9.17	17
PLAY	4	1	1	3.61	3.25
EXPERIENCES	4	4	4	8.94	20
DESIGN	5	2	2	5.83	6.2
**2018–2019 Period**					
	**Docs**	**h-index**	**q-index**	**q2-Index**	**Cites**
FLIPPED-TEACHING	3	0	0	0	0
SATISFACTION	16	4	6	6	2.94
INTRINSIC-MOTIVATION	16	5	10	8.66	6.62
IMPACT	14	4	6	6.63	3.29
GAMIFICATION	187	10	15	13.04	2.23
FLIPPED-CLASSROOMS	12	3	6	4.9	3.58
TECHNOLOGY	12	2	2	4.24	1.17
CLASSROOM	14	7	10	9.54	8.14
E-LEARNING	8	2	2	3.16	1
GAMES	9	2	4	5.1	3.22
VIDEO-GAMES	10	4	5	4.47	3.1
LEARNING	9	2	3	3.16	1.11
PEDAGOGY	5	1	1	1	0.4
ACTIVE-LEARNING	6	2	3	4	1.83
